# Unveiling the fungal frontier: mycological insights into inflammatory bowel disease

**DOI:** 10.3389/fimmu.2025.1551289

**Published:** 2025-03-26

**Authors:** Silan Chen, Meijing Yi, Xinying Yi, Yuxuan Zhou, Houpan Song, Meiyan Zeng

**Affiliations:** ^1^ School of Traditional Chinese Medicine, Hunan University of Chinese Medicine, Changsha, Hunan, China; ^2^ Hunan Provincial Key Laboratory of Traditional Chinese Medicine Diagnostics, Hunan University of Chinese Medicine, Changsha, Hunan, China

**Keywords:** inflammatory bowel disease, microbiology, fungi, pathogenesis, treatment

## Abstract

Inflammatory bowel disease (IBD) is a chronic recurrent gastrointestinal disease that seriously affects the quality of life of patients around the world. It is characterized by recurrent abdominal pain, diarrhea, and mucous bloody stools. There is an urgent need for more accurate diagnosis and effective treatment of IBD. Accumulated evidence suggests that gut microbiota plays an important role in the occurrence and development of gut inflammation. However, most studies on the role of gut microbiota in IBD have focused on bacteria, while fungal microorganisms have been neglected. Fungal dysbiosis can activate the host protective immune pathway related to the integrity of the epithelial barrier and release a variety of pro-inflammatory cytokines to trigger the inflammatory response. Dectin-1, CARD9, and IL-17 signaling pathways may be immune drivers of fungal dysbacteriosis in the development of IBD. In addition, fungal-bacterial interactions and fungal-derived metabolites also play an important role. Based on this information, we explored new strategies for IBD treatment targeting the intestinal fungal group and its metabolites, such as fungal probiotics, antifungal drugs, diet therapy, and fecal microbiota transplantation (FMT). This review aims to summarize the fungal dysbiosis and pathogenesis of IBD, and provide new insights and directions for further research in this emerging field.

## 1 Introduction

Intestinal microbiota, including bacteria, fungi, and viruses, represent a complex microbial ecosystem (1) and are an important part of human health. Although the fungi account for only about 0.1% of the intestinal microbial community (2), they play a decisive role in the dynamic balance of intestinal microbial composition and mucosal immune response, and have a disproportionate significance in the intestinal ecosystem (3). The abnormal changes of intestinal fungi are closely related to human digestive system diseases. In this review, we identify significant changes in intestinal microecology associated with common diseases and explore the association between fungi and inflammatory bowel disease (IBD) through basic and clinical research conducted by multiple research teams (4).

A number of studies (5–9) have shown that intestinal fungi can trigger inflammation by regulating the host's immune response, potentially increasing the host's susceptibility to various diseases and exacerbating IBD. It is worth noting that the abundance of fungi in the intestine is much lower than that of bacteria, and the instability of fungal structures makes it difficult to fully describe the composition of normal fungi (7). While traditional research has mainly focused on the effect of intestinal bacteria on IBD, research on intestinal fungi is mostly scattered and lacks systematic analysis and summarization.

This study explored the functions of fungi in various aspects of human gastrointestinal health, focusing on fungal microbiota imbalance, fungal-bacterial interactions, the influence of fungal metabolites, and fungal-based treatment strategies in IBD patients. By systematically integrating the understanding of the role of fungi in the pathogenesis of IBD, the important position of fungi in the intestinal ecosystem is revealed, addressing a significant gap in the field of intestinal microecology These findings will greatly enrich our understanding of the biological diversity and disease relevance of the human gut microbiome.

## 2 Intestinal fungal colonization in healthy people

Fungi are present in the GIT of all healthy individuals (10, 11), where they can influence the immune system (5) and produce health-affecting secondary metabolites (12, 13). Therefore, understanding a healthy mycobiota may aid in the identification of disease-contributing fungal species and allow a better determination of important fungal-bacterial relationships. The development of molecular biology techniques has significantly deepened the understanding of intestinal fungal diversity. The 18S rRNA gene sequencing provides a basic framework for fungal classification, while ITS2 sequencing has become a sensitive tool for detecting low-abundance fungi, with higher resolution than 18S rRNA gene sequencing, which can distinguish species and strain-level differences (10). However, this technique may omit some fungal information. In contrast, metagenomic sequencing can completely retain the genetic information of fungi by directly extracting the nucleic acids from the sample, allowing for the analysis of the functional genes within the microbiota and revealing the interactions between microorganisms, the environment, and the host (14).

**Figure 1:**
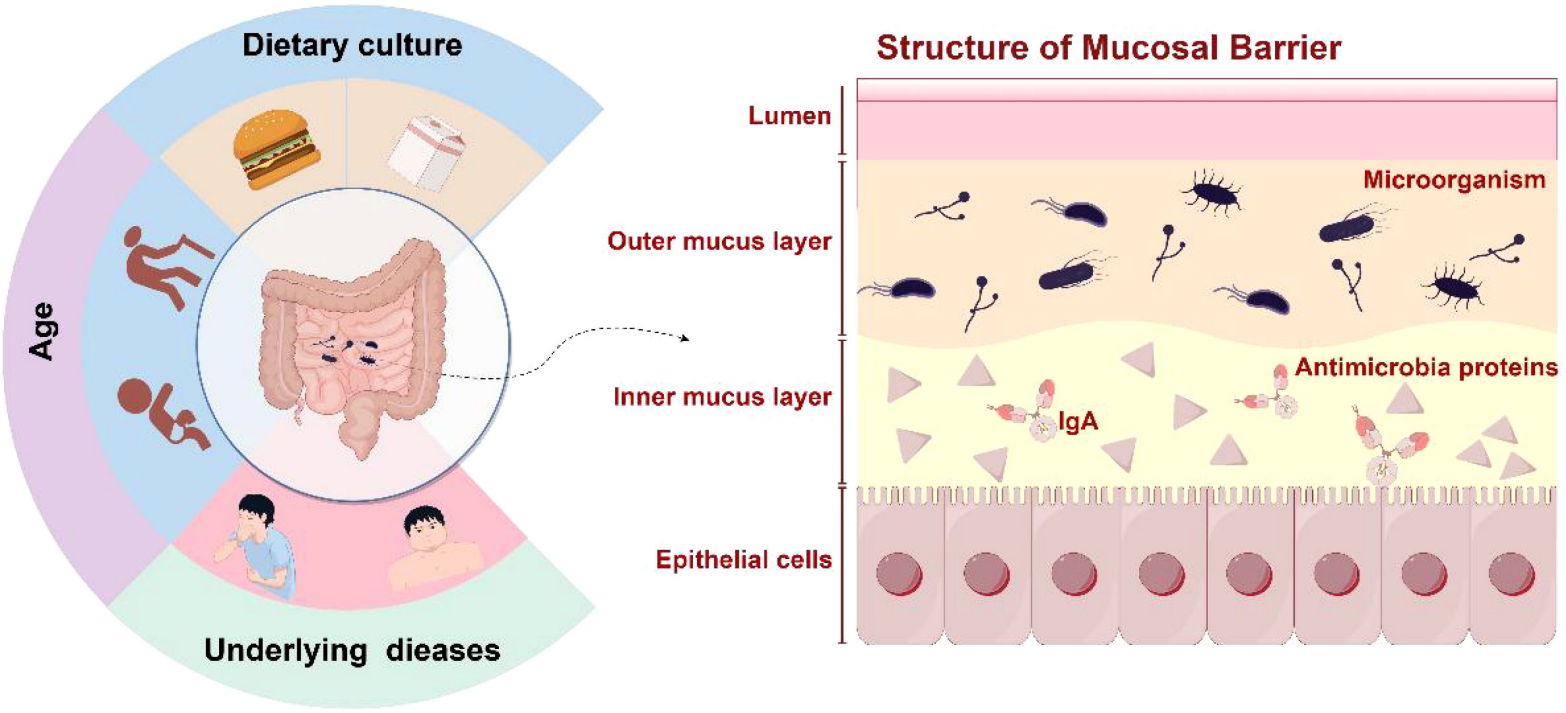
Factors influencing intestinal microbes. Microbial disorders alter the production of surface mucus, epithelial function, and intestinal immune defenses.

A number of studies have revealed the core composition of the human intestinal fungal group through high-throughput sequencing technology. Analysis of 317 fecal samples by the Human Microbiome Project (HMP) (10) showed that *Saccharomyces cerevisiae*, *Malassezia* spp., and *Candida albicans* were the core fungi with higher prevalence in healthy people. The Chinese Guangzhou Nutrition Health Study (GNHS) (15), based on a cohort of 1,244 middle-aged and elderly people, further expanded this conclusion by identifying 26 core fungal genera, including *Saccharomyces*, *Candida*, *Aspergillus*, and *Malassezia*, among which *Ascomycota* and *Basidiomycota* are dominant. Both studies confirmed the universality of *Malassezia* and *Candida*, suggesting that they may play a fundamental role in intestinal microecology. However, sequencing results may be affected by a variety of factors (**Figure 1**), such as potential diseases carried by volunteers (16), dietary culture (17), age (18), and other factors (19). The GNHS cohort study suggested that age may be a key factor in shaping individual differences in the intestinal fungal group. The abundance of *Aspergillus* and *Penicillium* in the body showed significant changes with age (20). Further analysis revealed that the abundance of *Bacillus and Trichoderma* spp. in the intestines of the elderly decreased significantly, while *Malassezia* was found to be enriched.

As mentioned above, there is a relatively stable core fungal community in the intestine of healthy individuals. Although its abundance is low, it maintains a dynamic balance under the normal regulation of the host's immune system. This homeostasis provides an important baseline for identifying disease-related fungal groups. Current studies have shown that the intestinal fungal communities are not only involved in the regulation of host metabolism but may also affect the disease process through a two-way interaction with the immune system. Research is urgently needed to construct a phylogenetic map of intestinal microecology and to analyze the colonization characteristics of fungi using functional metagenomics and metabolomics techniques. It is worth exploring:

(1) Fungi are transient microorganisms that exchange between the human gastrointestinal tract and the external environment. Genetic defects and environmental factors associated with IBD can induce the accumulation and penetration of pathogenic bacteria into intestinal tissues, thereby further promoting an imbalance in the microbial microbiota and inflammation (21). This colonization pattern suggests that disease status may significantly affect the intestinal residence characteristics of fungi, and the detection results can be used as an auxiliary index for disease diagnosis (4).
(2) Various types of fungi exist in the gastrointestinal tract of healthy humans, forming a stable core fungal community. When immune surveillance is impaired, these symbiotic fungi can become opportunistic pathogens, potentially leading to chronic inflammation by triggering TH1/TH17 immune responses or disrupting the Treg cell regulatory network (2, 22). In addition, the imbalance of fungi may disrupt the host's regulatory mechanisms for inhibiting inflammation and play an important role in the promotion of the pathogenesis of IBD (21). This mechanism provides a theoretical basis for the targeted regulation of fungal microbiota in the prevention and treatment of IBD.

Regardless of the answer, the role of the intestinal fungal colonization in the occurrence or treatment of diseases cannot be ignored. Analyzing the biodiversity of intestinal fungi and its correlation with diseases remains of great significance. Fungi play a key role in the ecological adaptation of the human gastrointestinal environment by participating in the degradation of fermentable substrates (i.e., polysaccharides, proteins, and lipids) and the biosynthesis of secondary metabolites (23). The *Pezizomycotina* species are involved in regulating a wider range of metabolic pathways than other fungi, with specific functions concentrated on secondary metabolism, as well as amino acid and carbohydrate transport and metabolism (4). Specifically, these fungi can produce a large number of plant cell wall-degrading enzymes (PCWDEs) for cellulose, hemicellulose, starch, and pectin, which indicates that the *Pezizomycotina* species may actively participate in the decomposition of plant polysaccharides in the intestines of certain organisms. In addition, *Saccharomycotina* species have significant proteolytic ability. It is worth noting that fungal secondary metabolites have unlimited potential in treating certain diseases and in synthetic biology. They are a prolific source of antibiotics (such as penicillin and cephalosporins) and immunosuppressive drugs (24), and they play a positive role in maintaining human intestinal health.

## 3 Inflammatory bowel disease

Inflammatory bowel disease (IBD) is a chronic autoinflammatory disease involving the gastrointestinal tract and extraintestinal organs. IBD, includes several clinical histologic variants, such as ulcerative colitis (UC), Crohn's disease (CD), and indeterminate colitis (IC) (21, 25–27). UC is characterized by chronic inflammation of the large intestine with aberrant activation of the immune system (28), manifesting as diffuse and persistent colonic inflammation extending proximally from the rectum. About 40-70% of UC patients are characterized by mild inflammation of the upper gastrointestinal tract (29).CD commonly involves the terminal ileum and colon and may present as an inflammatory, penetrating, stenotic, or mixed phenotype (30). While the inflammation and damage in UC are limited to the mucosa, CD may be a transmural process (30). The latest global epidemiological data show that the prevalence of IBD has reached 0.5-1.5% in European and American countries (31), while the annual incidence in Asia is increasing to a rate of 0.3-0.5%. It is expected that the number of global IBD patients will exceed 8 million by 2025 (32). IBD not only causes severe physiological dysfunction, such as diarrhea, rectal bleeding, and abdominal pain (33–35), but also significantly increases the risk of colorectal cancer.

The pathogenesis of IBD involves three main factors: individual and genetic susceptibility, the gastrointestinal microbial community, and immunological characteristics of the gastrointestinal mucosa (36). The primary pathogenesis of the two forms of IBD (UC and CD) is related to the imbalance of the symbiotic microbiota living in the intestine. When the intestinal barrier function is impaired, symbiotic bacteria and their metabolites penetrate the mucosal layer, triggering the abnormal activation of dendritic cells and leading to a cascade release of pro-inflammatory factors such as IL-6 and TNF-α, which form a chronic inflammatory cycle (21, 37, 38). In addition, active intestinal inflammation is accompanied by excessive production of reactive oxygen species (ROS) (39), which leads to oxidative damage, participates in the activation of the intestinal immune system, and exacerbates the inflammatory response (40).

The current clinical treatment for IBD still faces multiple challenges: traditional 5-aminosalicylic acid (5-ASA) preparations are only effective for mild UC (41, 42), immunosuppressants (such as azathioprine) are slow to take effect, and there is a risk of bone marrow suppression (43, 44). Although biological agents (such as anti-TNF agents) can quickly induce remission, about 30% of patients develop primary drug resistance (45). Additionally, long-term use may increase the risk of opportunistic infections, such as tuberculosis and lymphoma. Although surgery (such as total colectomy) can cure UC, the postoperative quality of life of patients is reduced, and the recurrence rate of CD is as high as 50% (46). Therefore, new therapies such as targeting intestinal microecology and regulating immune response have emerged as research hotspots. However, their long-term efficacy and safety still need to be further verified.

## 4 IBD and fungal disorders

Alterations in the gut microbiota play a key role in the onset, progression, and severity of IBD. Diet-related factors, including childhood exposure to antibiotics and dietary emulsifiers (47–49), as well as the Western diet, which contains several high-fat foods and refined sugars, participate in IBD pathogenesis (50, 51). These factors can lead to ecological disorders and are closely related to intestinal inflammation. Intestinal microbes establish a link between the environment and the intestine. Diverse microbial ecosystems inhabit the human body and play a critical role in maintaining host health, including various fungal species known as "fungalome" (10). The study of intestinal fungal microbiota and its correlation with gastrointestinal diseases, especially IBD, has attracted much interest in recent years (**Table 1**). These studies have provided a new perspective on the etiology and non-invasive diagnosis of gastrointestinal diseases (27, 52). Although research on the fungal group is still in its infancy stage, it is rapidly gaining recognition for its potential role in human disease.

**Table 1:** Changes in fungal microbiota in IBD patients.

Disease	Sample	Fungal changes	Reference
Crohn’s disease	Feces	*Candida albicans*↑	(54, 57)
		*Candida↑*, *Entyloma↑*, and *Trichosporon↑* *Hanseniaspora↓*, *Hypsizygus↓*, *Wallemia↓*	(56)
		*Candida↑* *Aspergillus↓*, *Penicillium genera↓ unclassified Sordariomycetes↓*	(164)
	Mucosa	*Cystofilobasidiaceae family↑*, *Dioszegia genera↑*, *Candida glabrata↑*	(62)
		*Ascomycetes↑*, *Basidiomycetes↓* *Malassezia restricta↑*, *Cladosporium↑* *Fusarium↓*	(165)
		*Debaryomyces hansenii↑*	(63)
Ulcerative colitis	Feces	*Dipodascus↑*, *Entyloma↑*	(56)
		*Candida↑*	(4, 9)
	Mucosa	*Scytalidium↑*, *Morchella↑*, *Paecilomyces↑* *Humicola↓*, *Wickerhamomyces*↓	(166)
		*Candida↑* *Saccharomyces↓*	(8)
IBD	Feces	*Candida albicans↑* *Saccharomyces cerevisiae↓*	(54)
		*Shannon diversity↓*	(4)

↑ indicates a significant increase in fungal abundance/activity.

↓ indicates a significant decrease in fungal abundance/activity.

The gastrointestinal tract contains highly diverse fungi, which is the most studied fungal niche in humans. A total of 66 genera (divided into about 180 species) have been identified via ITS amplicon sequencing and whole genome sequencing of marker genes in DNA purified from feces. *Ascomycota* and *Basidiomycota* are dominant in the fungal group (53–55). Notably, *Candida*, *Saccharomyces*, and *Cladosporium* are the most abundant fungal genera in healthy individuals (38). In a small sample study in Japan (56) with 18 UC patients, 20 CD patients, and 20 healthy controls, sequencing results showed that there was no change in the abundance ratio of *Ascomycota* and *Basidiomycota* during IBD remission. However, another study (54) showed that the expansion of *Basidiomycota* and the reduction of *Ascomycota* occur during the onset of IBD. The study investigated the imbalance of fungal microbiota in IBD patients and found that the fungal burden of colonic mucosa increases during CD and UC (57, 58). Besides, several studies have shown that the abundance of *C. albicans* increases in IBD patients (54, 57). Furthermore, the abundance of *C. albicans* and *Candida parapsilosis* is increased in the inflammatory mucosa of CD patients (59). Meanwhile, the incidence and abundance of culturable *C. albicans* are increased in the feces of IBD patients (37, 60). Furthermore, the proportion of *Candida tropicalis* (61) is increased in the intestinal tract of CD patients and is positively correlated with the incidence of familial CD. Similarly, the abundance of *Candida glabrata* (62) and *Debaryomyces hansenii* (63) also increases in CD patients, especially in the inflammatory mucosa. Fungal dysregulation occurs in IBD patients, indicating that fungi play an indispensable role in maintaining the balance of intestinal microbiota.

## 5 Interaction of fungi with the host immune system

Fungi are common inhabitants of the intestinal barrier surface. The stability of the fungal community is essential for maintaining intestinal health. Abnormal changes in the fungal community(dysbiosis) can affect surface mucus production, epithelial function, and intestinal immune defense, thereby damaging the intestinal barrier and increasing its permeability (7, 64). Furthermore, changes in intestinal barrier permeability can allow opportunistic fungi or their components to enter the circulatory system (65, 66). At present, research on the interaction between specific fungi and the host immune system not only focuses on *C. albicans* (67) and *S. cerevisiae* (54), but also explores other fungi, such as *Cryptococcus neoformans* (68, 69) and *Aspergillus fumigatus* (70). However, there is still a lack of understanding regarding how the host immune system timely and accurately recognizes various mechanisms of fungal invasion and colonization (71).

**Figure 2:**
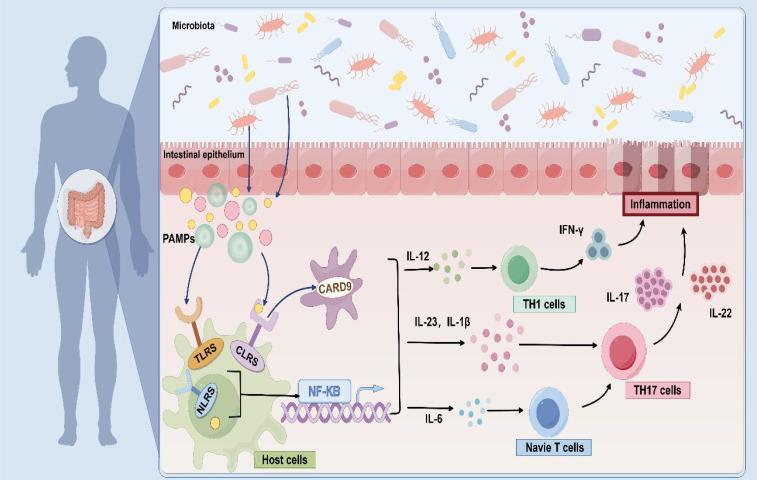
Fungal-induced immune response. As pathogen-associated molecular patterns (PAMPs), fungal cell wall components are recognized by pattern recognition receptors (PRRs), and which activate various signal cascades by inducing CARD9 to release a variety of cytokines, such as IL-12, IL-6, IL-1β, IL-23, and so on. These cytokines further promote TH1 and TH17 cell responses and initiate immune function to modulate fungal immune recognition.

Fungi can induce unique local and systemic cytokine signals through multiple pathways. Dectin-1, CARD9, and IL-17 signaling pathways may be immune drivers of fungal dysbacteriosis in the development of IBD and participate in the regulation of the stability of the intestinal internal environment. The signaling cascade suggests that fungal dysbiosis can activate host protective immune pathways related to epithelial barrier integrity (72), release various pro-inflammatory cytokines to trigger inflammatory responses, thus promoting the occurrence and development of IBD. As an important part of the immune system, macrophages can recognize various complexes, such as β-glucan on the fungal cell wall (66, 73), then activate the body's innate and acquired immune response through the release of a variety of cytokines (74). Macrophages can also trigger inflammatory responses associated with IBD (**Figure 2**).

Phagocytes express and activate members of the C-type lectin receptors (CLRs), Dectin-1, Dectin-2, and Mincle (75), as well as pattern-recognition receptors (PRRs) (76), such as Toll-like receptors (TLRs) (77) and Nod-like receptors (NLRs) during this immune response, which have secondary roles on the surface of the host cells. Taking *C. neoformans* as an example, its capsular polysaccharide can be recognized by TLR4, thereby initiating an immune response (78). Additionally, the cell wall components of *A. fumigatus* can activate the NLRP3 inflammasome and cause inflammation (79). Phagocytes then recognize pathogen-associated molecular patterns (PAMPs) and signaling, inducing a signaling cascade via caspase-associated recruitment domain 9 (CARD9) (80) and nuclear factor-κB (NF-κB) to produce pro-inflammatory cytokines in response to innate immunity, and dendritic cells to release TH17 and TH1 in response to acquired immunity (81).

The pro-inflammatory environment further drives the differentiation of TH17 cells, closely linking the immune system with intestinal tissue and becoming central to the fungal immunopathology of IBD. The Dectin-1-CARD9 axis specifically induces TH17 cells to produce IL-17 and IL-22 by regulating the secretion of IL-23 and IL-1β (2, 74, 82). It's important to highlight that there are differences in the regulation of type 17 immunity by different fungi. This immune response is particularly significant when *C. albicans* is present; its β-glucan can activate dendritic cells through Dectin-1, promote the secretion of IL-12 and TNF-α, and enhance the pathogenicity of TH17 cells (83). *S. cerevisiae* has been reported to promote the induction of Treg cells through the Dectin-1-Cyclooxygenase-2 (COX-2) signal transduction axis and to directly inhibit the production of IFN-γ, which differentiates TH1 cells, through a TLR2-dependent mechanism, thereby reducing the inflammatory response in a dual manner (84, 85). *Malassezia* can aggravate skin and mucosal inflammation in patients by activating the IL-23/IL-17 axis (86). This two-way regulation mechanism suggests that targeting the fungi may become a new direction for IBD treatment. For example, Dectin-1 and Dectin-2 double gene knockout can effectively prevent intestinal inflammation by changing the intestinal microbiota of bacteria (87).

## 6 Pathogenic mechanism of fungi

The previous section discusses the abnormal activation of the host immune system by intestinal fungi, which leads to an inflammatory response. Therefore, it is necessary to explain the process of fungal pathogenesis. Fungi can elicit immune responses from susceptible hosts through various mechanisms, including mycelium formation, secretion of fungal cytolytic peptide toxins, and secretion of EVs, thus leading to the development of diseases.

### 6.1 Candidalysin

Candidalysin is one of the fungus-derived metabolites that plays a key role in host-microbe interactions. Candidalysin is potentially responsible for invasive mucosal infections and tissue damage, which can impact IBD (88). Candidalysin activates epithelial pro-inflammatory responses via two mitogen-activated protein kinase (MAPK) signaling pathways (p38/c-Fos transcription factor and MEK/ERK/MKP1) and induces downstream immune responses (89, 90), including neutrophil recruitment and innate type 17 immunity (91). MAPK signaling is a "danger-response" pathway that releases large amounts of granulocyte-macrophage colony-stimulating factor (GMCSF), triggering the recruitment of neutrophils, macrophages, and other immune cells, thus inducing local inflammatory response (6).

The epidermal growth factor receptor (EGFR) may be activated through the shedding of endogenous ligands, such as amphiregulin (AREG), epigen (EPG), and epiregulin (EREG) from epithelial cells through Ca^2+^ in-flow and Matrix metalloproteinases (MMPs), thereby activating downstream mitogen-activated protein kinase signaling (c-Fos transcription factor and MKP1), thus activating epithelial immunity (6).

Studies have shown that Candidalysin can also induce MAPK signaling through MEK1/2 and ERK1/2, leading to the activation of the AP-1 transcription factor c-Jun/c-Fos and up-regulation of the secretion of neutrophil-collecting chemokine (C-X-C motif chemokine ligand 8, CXCL8). This results in the recruitment of several neutrophils to the site of infection, triggering excessive inflammation and causing vascular endothelial cell injury (88).

### 6.2 Extracellular vesicles

Extracellular vesicles (EVs) produced by eukaryotes, archaea, and bacteria are lumen-containing spheres with a diameter of 20-500 microns. EVs consist of a lipid bilayer containing lipids, proteins, polysaccharides, pigments, and nucleic acids (92). The release of EVs is an alternative transport mechanism for macromolecules across the fungal cell wall (93, 94). The vesicles move outward across the cell wall, eventually reaching the extracellular environment (95–100). Besides, the impact of EVs in pathological and physiological processes is under investigation (101–104). The cell wall and capsule structure are major pathogenic factors in most fungal species because they protect cells from unfavorable environmental conditions and host immune factors, ensuring their survival (105). Therefore, the involvement of fungal EVs in the biogenesis and maintenance of capsule and cell walls can be considered a pathogenic mechanism. Recent studies have shown that these vesicles are associated with key virulence factors. Besides, EVs are biologically active, causing host cell death (106) and triggering an immune response (107, 108).

**Figure 3:**
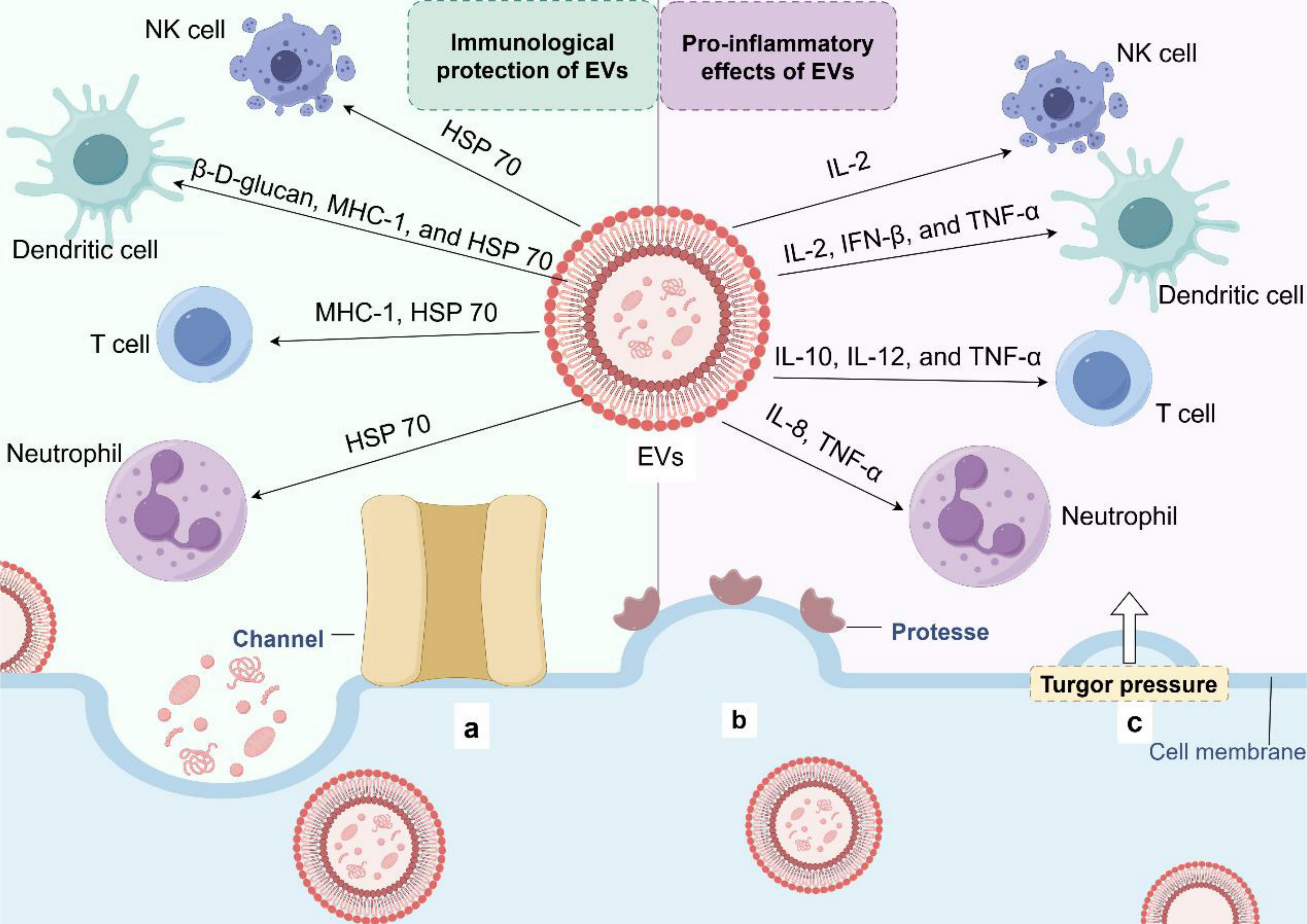
Extracellular vesicle release and immune potential. **(a)** Protein channels may modulate the transport of EVs in the extracellular environment. **(b)** The 'loose' effect of cell wall-modifying enzymes may promote EV release. **(c)** EVs pass through the wall under the influence of turgor pressure.

EVs from several pathogenic fungi play a “double-edged sword” role in the regulation of the immune system (109) (**Table 2**, **Figure 3**). EVs play an important role in fungal infection and dissemination, acting as mediators of cellular communication, thus influencing interactions between fungi and host cells (macrophages (110, 111) and dendritic cells (112, 113)) and transmitting virulence factors to the host. EVs increase cryptococcal passage across the blood-brain barrier and allow cryptococcal-derived EVs to accumulate in fungal brain damage, suggesting a novel role for EVs in fungal pathogenesis (114). Fungal EVs can directly influence biofilm production and regulate dimorphism in *C. albicans*. Studies have shown that yeast-produced *C. albicans* EVs can induce filamentation and growth of *C. albicans* (115). EVs reduce the intracellular ROS and apoptosis of *C. albicans* by activating the L-arginine/NO pathway, thus enhancing the damage to the host cell caused by *C. albicans* (106).

**Table 2:** The bidirectional immunoregulatory effect of EVs.

Types	Functions	
	Pro-inflammatory	Immune potential
*Candida albicans* EVs	In the early stage of *C. albicans* infection, blood monocytes released EVs with a large number of functional important microRNAs, which induced significant growth and hyphal formation of *C. albicans* (167).	Fungal EVs from *C. albicans* activate humoral immune responses, which decreases the fungal load in immunosuppressed mice and produce immune protection (168).
	EVs have an interchangeable role in biofilm drug tolerance and dispersion, and are the core of biofilm pathogenicity (169).	EVs decrease the adhesion and invasion ability of *C. albicans* by inhibiting the differentiation of yeast into hyphae, which reduces fungal virulence (108).
	EVs inhibit ROS production by activating the L-arginine / nitric oxide pathway to reduce apoptosis and promote the growth of *C. albicans* (106).	EVs derived from *Candida haemulonii* var. *vulnera* have immunogenicity and stimulate bactericidal function in mouse macrophages by stimulating the production of ROS (170).
	EVs activate inflammatory responses in polymorphonuclear cells and RAW264.7cells *in vitro* (171).	*In vivo* experiments demonstrated that EVs inoculated with *C. albicans* provided a protective response mediated by TLR4-dependent activation in mice infected with fungi (172).
*Cryptococcus neoformans* EVs	EVs regulate the generation of inflammatory cytokines through bone marrow-derived dendritic cells and macrophages (173).	Protective antigens are present on the surface of EVs, producing a strong antibody response in mice and have the potential as a vaccine (174).
*Aspergillus flavus* EVs	EVs stimulate macrophages to generate inflammatory mediators, enhance the phagocytosis and killing effect of macrophages, and promotes M1 macrophage polarization *in vitro* (175).	EV proteome cargo in fungal endophthalmitis has host immune-related proteins, such as complement component 8α, acting as an crucial antibacterial immune effector (176).
*Talaromyces marneffei* EVs	The RAW264.7 macrophages interact with EVs upregulating IL-1β, IL-6, IL-10, and TNF-α levels, promoting inflammatory response (177).	EVs can effectively regulate the function of macrophages, thereby activating these innate immune cells and enhancing their antibacterial activity (178).

### 6.3 Fungal-bacterial interactions

**Figure 4:**
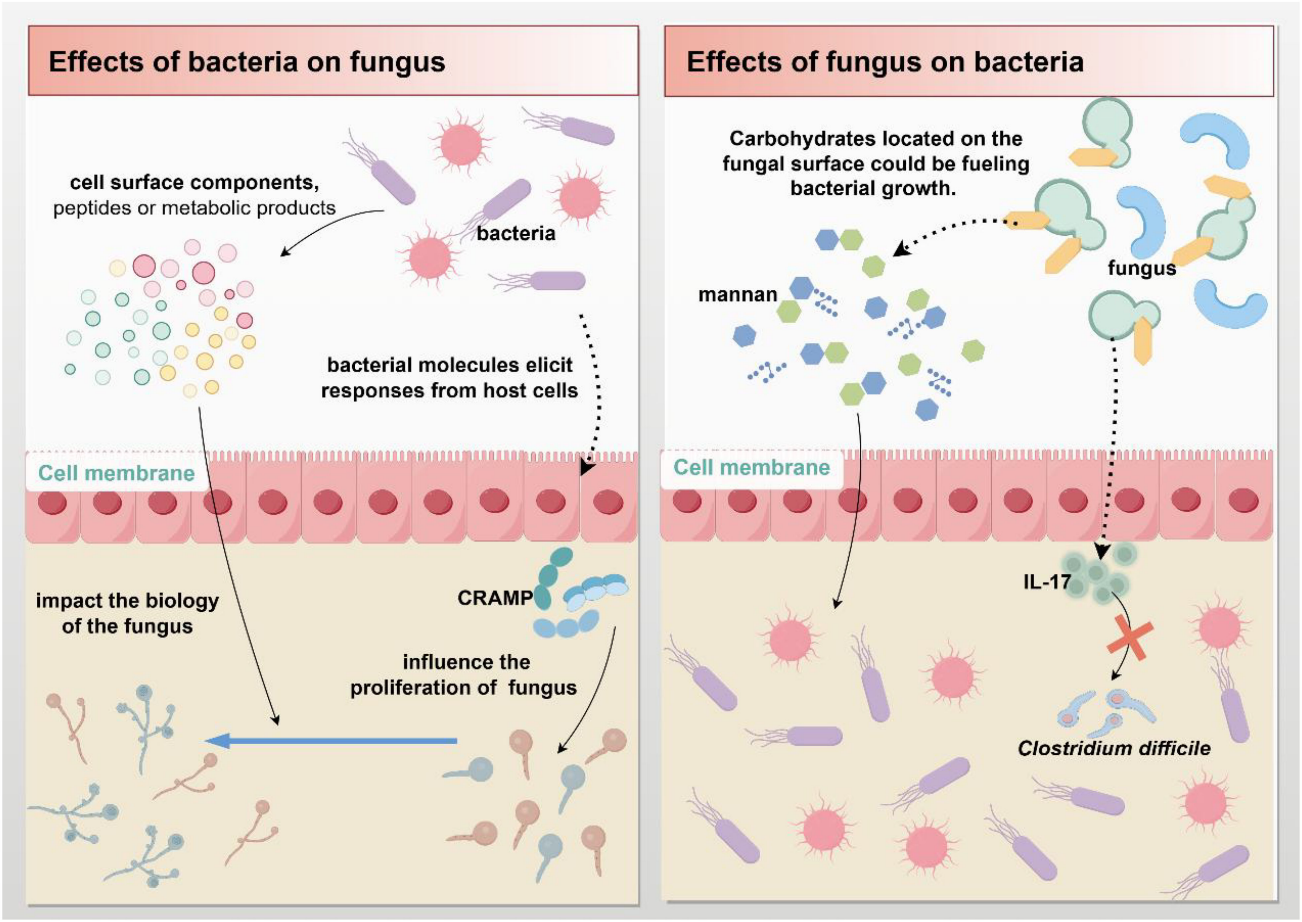
Fungal-bacterial interactions. Gut microbes can directly or indirectly influence each other's biological processes by secreting molecules (such as cell surface components, peptides, and metabolites) or triggering immune responses from host cells.

Fungi or any other microbial group can function alone in a crowded ecosystem, such as the human gut (116). Dysbiosis of the intestinal microbiota, a collective feature of complex interactions between prokaryotic and eukaryotic microbial communities, can affect immunity and render normally benign symbiotic organisms pathogenic (117). The importance of these fungal-bacterial interactions in human health and disease has attracted much attention in recent years. Intestinal fungi and bacteria interact with each other in various ways, including chemical or physical interactions, competition for nutrients or adhesion sites, and hybrid biofilm formation (61) (**Figure 4**).

#### 6.3.1 Bidirectional action of bacteria on fungi

Studies have shown that gut bacteria can affect the proliferation of *C. albicans* or other fungi (or influence any other fungal trait) through at least two primary mechanisms: First, an indirect pathway involving bacterial molecules (or microorganisms) triggering host cell responses on fungi (116). Bacteria limit fungal proliferation by stimulating the production of intestinal mucosal immune defenses, particularly the antimicrobial peptide cathelicidin-related antimicrobial peptide (CRAMP) (118). Second, the release of molecules, such as cell surface components, peptides, or metabolites, which can directly influence fungal biology (116). Bacteria can promote *C. albicans* infection and increase the risk of disease through released peptidoglycan subunits (119–121). An *in vitro* co-culture study found that *Escherichia coli* can produce a soluble factor that kills *C. albicans* in a magnesium-dependent manner (122).

Meanwhile, studies have shown that pathogenic bacteria in the gut can produce fungal modifying compounds and secreted enzymes, which affect the composition of the intestinal fungal community (123). *Streptococcus mucosus* produces two secreted proteins, Tfe1 and Tre2, which induce antifungal effects by reacting with *C. albicans* and yeasts (124). Peptides and toxins secreted by the bacteria may also act on nearby cells, thus inhibiting fungal infection and colonization. *Enterococcus faecalis* is a Gram-positive commensal bacterium that parasitizes the human gastrointestinal tract. *E. faecalis* can produce an active peptide containing 68 amino acids (EntV), which can inhibit the formation of *C. albicans* hyphae and prevent biofilm formation (125, 126).

#### 6.3.2 Bidirectional action of fungi on bacteria

Fungi and bacteria have similar morphologies on the surface of the intestinal mucosa and can interact with each other. Dysregulation of the fungal microbiota in the intestine often leads to changes in the composition of the bacterial microbiota (117). *C. albicans* can increase resistance of *Staphylococcus aureus* to antibiotics by secreting farnesol (127, 128). Also, carbohydrates on fungal surfaces can promote bacterial growth. The simpler sugars produced by the digestion of mannan by *Bacteroides* spp. enzymes promote the proliferation of *Bacteroides* spp (129). Meanwhile, fungi are resistant to bacterial infections. A study showed that *C. albicans* can protect mice against lethal *Clostridium difficile* infection, partly through the promotion of the production of the pro-inflammatory cytokine IL-17 (130).

These findings indicate that intestinal fungi closely interact with bacteria. This interaction may play an important role in the development of IBD. However, studies on the interactions between intestinal fungi and bacteria in IBD patients are still in the infancy stage, and the specific modes of interaction are unclear. Therefore, more in-depth studies are needed to reveal the physical and chemical interactions between intestinal fungi and bacteria in IBD.

## 7 Fungal-based treatment

The previous sections expound on the role of fungi in various aspects of human gastrointestinal health, focusing on fungal-host immune system interactions, fungal-bacterial interactions, and the influence of fungal metabolites. The following section focuses on exploring the treatment of IBD by targeting fungi based on the aforementioned pathogenic mechanisms.

**Figure 5:**
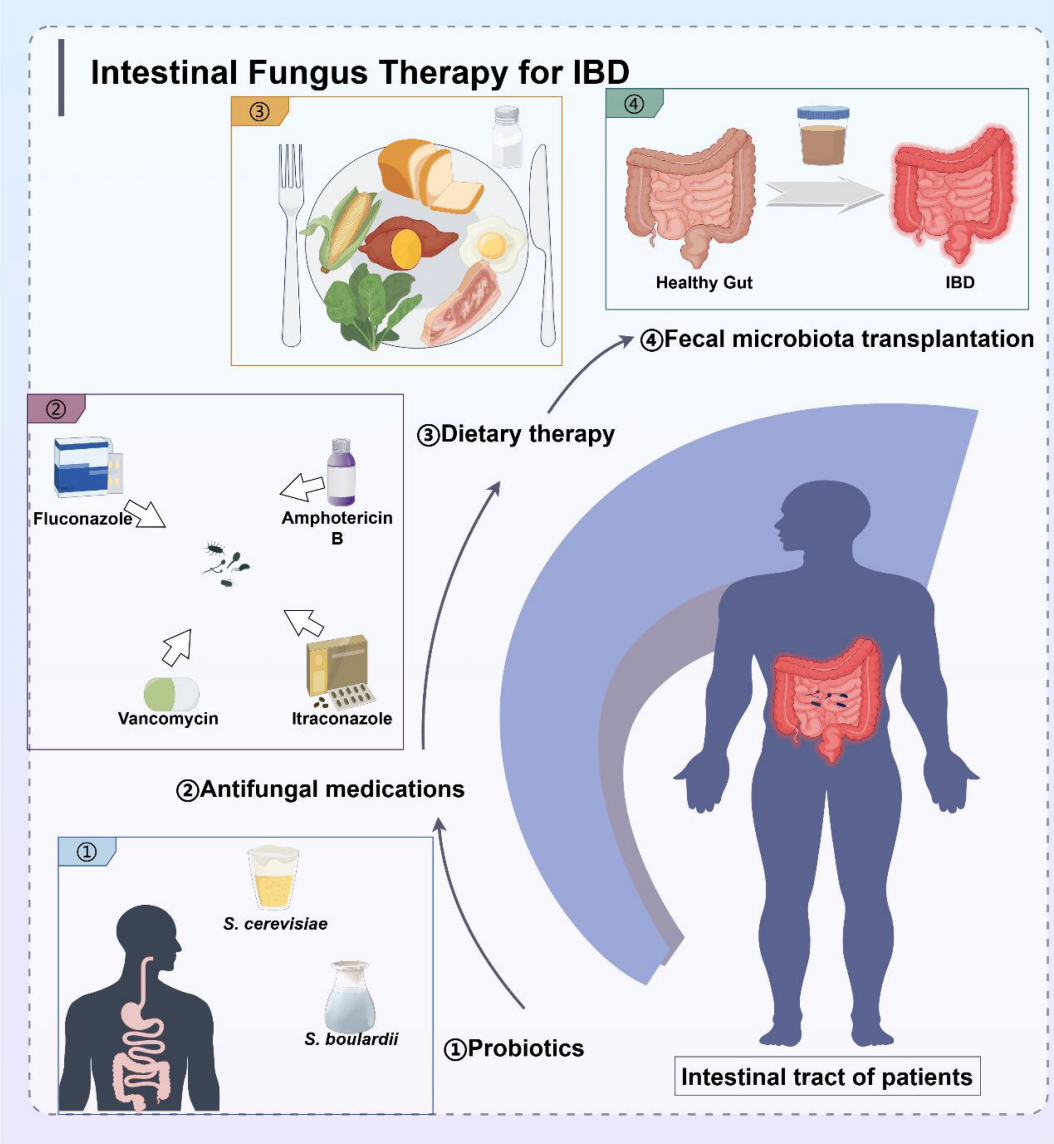
Treatment strategies for IBD targeting intestinal microbiota, including probiotics, antifungal medications, dietary therapy, and fecal microbiota transplantation.

Standard clinical treatment for IBD consists of drugs that modulate the inflammatory pattern of gastrointestinal tract, including mesalazine, azathioprine, anti-tumor necrosis factor (TNF), and glucocorticoids (131). However, these drugs have serious side effects. Besides, some patients require higher doses throughout the treatment. Although the exact etiology of IBD is unknown, the critical role of gut fungi in the development and persistence of IBD highlights the importance of fungal microbiota-host interactions in health and disease. Therefore, targeting the intestinal fungal group and its metabolites may be a novel strategy for IBD treatment. The gut fungal microbiota affects the host by modulating physiological, pathophysiological, and immune processes. Experimental animal studies and clinical data have demonstrated that gut mycobiome ameliorates inflammation, highlighting its potential as a therapeutic strategy for the treatment of inflammatory diseases. Probiotics (132), antifungal medications, dietary therapy, and fecal microbiota transplantation (FMT) are the most common IBD treatments (**Figure 5**). UC typically involves a local mucosal immune response, whereas CD is mostly a transmural process that engages a broader cross-level immune response. Due to these different immune mechanisms, the correlation between fungi and bacteria in UC and CD patients varies significantly. Specifically, the positive and negative correlations between bacteria and fungi in UC patients are stronger than those in CD patients, which also leads to differences in the treatment methods for these two diseases. Antifungal therapy and probiotic therapy are more conducive to the recovery and healing of colon injury of UC, while most patients with CD eventually require surgical treatment, with fungal-based treatment playing a supportive role.

### 7.1 Probiotics

The beneficial effects of probiotics have been clearly demonstrated to prevent the recurrence of pouchitis in patients after colectomy (133), and certain specific probiotics also seem to directly regulate intestinal pain. Although bacteria, such as *Lactobacillus* spp. and *Bifidobacterium* spp., are the most widely used probiotic microorganisms, some fungi can become probiotics due to their health benefits (134). Fungal probiotics possess several favorable properties: First, fungi are naturally resistant to antibiotics and can thus be used in combination with them to treat certain diseases (135). *S. cerevisiae* is the most studied fungal probiotic and probably the most promising fungal probiotic species. The intervention of *S. cerevisiae* can increase the diversity of intestinal microbiota in mice with UC, improve the microbiota structure, decrease the relative abundance of the harmful bacteria *Escherichia-Shigella* (136) and *Turicibacter* (137), and increase the relative abundance of the beneficial bacterium *Lactobacillus* (136). Additionally, *S. cerevisiae* can significantly improve the physiological condition of mice with colitis, alleviate the histopathological damage of the colon, and enhance the intestinal mucus layer (136). *S. cerevisiae* can alleviate UC by up-regulating the expression of tight junction protein-related genes in Caco-2 cells (136, 138) and down-regulating the expression of pro-inflammatory cytokine-related genes (136, 139, 140). Kunyeit et al. examined the effects of food-derived *S. cerevisiae*, as a probiotic, on cell morphology and filament formation (141). The results showed that *S. cerevisiae* significantly inhibited the mycelial development of *Candida tropicalis* and *Candida parapsilosis*, and could better resist pathogenic damage from fungi.

*Saccharomyces boulardii* is another probiotic fungus with ecological regulatory effects that has been widely used for UC treatment. Sougioultzis et al. (142) found that *S. boulardii* induces anti-inflammatory activity through the production of a low molecular weight soluble substance that inhibits NF-κB activation in monocytes and intestinal epithelial cells. Animal experiments have shown that *S. boulardii* solution can significantly alleviate intestinal inflammation in rats with 2, 4, 6-Trinitrobenzenesulfonic acid (TNBS)-induced colitis (143). *In vitro* explant experiments showed that *S. boulardii* recovers the tight junctions between intestinal epithelial cells, restores and strengthens the intestinal barrier function by enhancing E-cadherin expression via RAB11A-dependent endosomal recycling (144).

### 7.2 Antifungal medications

Antimicrobial treatment reduces bacterial abundance and can indirectly affect fungal microbiota. A study has shown that in the case of intestinal inflammation, the positive and negative effects of fungi depend on the presence of *Enterobacteriaceae* bacteria. Specifically, mice treated with vancomycin (targeting Gram-positive bacteria) were completely protected from colitis, while mice treated with colistin (targeting *Enterobacteriaceae*) retained the colitis phenotype but were no longer affected by fungal administration (145). Fluconazole, an antifungal drug, is commonly used to treat fungal infections such as candidiasis and candidemia in patients with immunosuppressed IBD. In a study involving 89 UC patients, 20 patients with high fungal colonization showed a significant decrease in the disease activity index, as reflected by clinical, endoscopic, and histological criteria, after 4 weeks of fluconazole treatment compared to the placebo group and the probiotic Lacidofil group (36). Studies have shown that amphotericin B and itraconazole can be used to treat infections caused by *Histoplasma capsulatum*, significantly reducing or completely eradicating the pathogen (146, 147). The incidence of *Histoplasma capsulatum* infection is high in patients with colitis. Itraconazole can alleviate the symptoms of IBD, especially in UC patients, can achieve both clinical and endoscopic remission (148). The above drugs restore the balance of intestinal microorganisms through antifungal drug treatment, which proves the feasibility of targeted fungal treatment for IBD.

### 7.3 Dietary therapy

Studies have demonstrated that diet can shape the composition of the gut microbiota. Gut microbes utilize diet-derived nutrients to grow and colonize the gut (149). In contrast, host cells use microbial metabolites as substrates for energy production and immunomodulators to maintain gut homeostasis. This symbiotic relationship between the gut microbiota and the host is critical to the human health (21). Dietary therapies can independently influence the composition of microbiota to reduce inflammation, and changes in the composition of a single food group may have profound effects. Switching from a plant-based to an animal-based diet radically alters bacterial taxa and metabolism, causing alterations to bile acid and sulphide metabolism, both of which influence the development of UC (150). Consumption of certain diets, such as Western diets characterized by high fat and low fiber may induce dysbiosis of the gut microbiota, disrupting intestinal homeostasis and promoting intestinal inflammation (151). A study comprising 98 healthy adults (152) reported that the abundance of Candida was positively associated with a carbohydrates-rich diet and negatively associated with a diet rich in protein, fatty acids, and amino acids (153). The abundance of Aspergillus was negatively associated with recent intake of short-chain fatty acids (152). Dietary fiber is broken-down to short-chain fatty acids by gut microbes, providing a protective effect. A clinical study (154) showed that the intake of fruits and vegetables was negatively correlated with the risk of IBD and its subtypes. Additionally, dietary fiber intake was negatively correlated with the incidence of CD, but not with UC. *Herba houttuyniae* is a potential dietary intervention, and the extract Sodium houttuyfonate induces β-glucan production and stimulates intestinal macrophages to clear colonized *C. albicans* (155, 156). Coconut oil reduces gastrointestinal colonization by the opportunistic pathogen *C. albicans* and has anti-inflammatory, hypolipidemic, and antidiabetic effects (157).

### 7.4 Fecal microbiota transplantation (FMT)

Fecal microbiota transplantation (FMT) is a strategy used to transfer feces from a healthy donor to the gut of a patient with IBD (158), with the aim of restoring microbial homeostasis. To date, some patients with IBD treated with FMT show variable response, with about 30% of patients with UC achieving clinical remission (159). The therapeutic efficacy of FMT has been attributed to gut bacteria for a long time, however, in recent years the important role of gut fungi in FMT outcomes has also been revealed (160).

Patients with IBD are susceptible to *C. difficile* infections and often exhibit increased abundance of *C. albicans* (161). Studies have shown that FMT modulates the intestinal fungal composition of patients (162) and is particularly effective in UC patients with high abundance of intestinal *Candida*. FMT treatment decreased *C. albicans* abundance in patients with UC and alleviated IBD severity by inhibiting the pro-inflammatory immune response induced by intestinal fungi (162). However, only a small group of patients showed good response to FMT, and further studies are needed to explore whether other intestinal fungi may influence its efficacy. The latest research (163) has demonstrated that *C. albicans* exhibits antagonism with *Pseudomonas aeruginosa*, *Lactobacillus*, and *Salmonella*, suggesting that it may be a promising treatment for *C. albicans*.

However, the role of intestinal fungi in FMT is not fully understood with some questions remaining to be unanswered (158). Further studies should explore the adverse effects of FMT and develop strategies to prevent damage to the host (72). For example, the presence of *C. albicans* in the feces can inhibit the clearance of *C. difficile* when fecal fungal transplants are administered to treat *C. difficile* infections. In addition, complex fungal-bacterial interactions may decreased the efficacy of FMT.

## 8 Conclusions

This paper analyzes the relationship between dysfunctional fungal microbiota and inflammatory bowel disease, as well as the pathogenic mechanisms of intestinal fungi. Although there are significant differences in the experimental studies of intestinal fungal colonization among healthy individuals in various countries and regions, there are commonalities in the dynamic changes associated with IBD. Fungal-host immune interactions and fungal-bacterial interactions are considered important mechanisms by which the intestinal fungal modulates IBD. Fungi can induce unique local and systemic cytokine signals through various mechanisms. TH17 cells play a critical role in linking the immune system to intestinal tissues, helping to regulate specific symbiotic bacteria and fungi, which is crucial for the pathogenesis of fungal infections. By understanding the fungal-bacterial-host interactions, we can identify patients at risk of IBD and develop customized interventions for the microbiome to reduce the serious side effects of traditional IBD treatments. Nevertheless, there are some limitations that need to be mentioned. Firstly, although genome sequencing technology was employed to identify key fungal species, there is currently little data for describing the fungal microbiome, especially those involved in the development of IBD. Secondly, the impact of intestinal fungal-bacterial associations in IBD are not fully understood. To increase our understanding of the specific role of microbiome in health and disease, more studies are advocated.
